# Liposomal formulations of carboplatin injected by convection-enhanced delivery increases the median survival time of F98 glioma bearing rats

**DOI:** 10.1186/s12951-018-0404-8

**Published:** 2018-10-05

**Authors:** Minghan Shi, Malathi Anantha, Mohamed Wehbe, Marcel B. Bally, David Fortin, Laurent-Olivier Roy, Gabriel Charest, Maxime Richer, Benoit Paquette, Léon Sanche

**Affiliations:** 1 Department of Radiation Oncology, The Second Affiliated Hospital of Zhejiang University, School of Medicine, Hangzhou, China; 2 Experimental Therapeutics, British Columbia Cancer Agency, Vancouver, BC Canada; 3 Centre for Drug Research and Development, Vancouver, BC Canada; 4 Department of Surgery, Division of Neurosurgery, Centre Hospitalier Universitaire de Sherbrooke, Sherbrooke, QC Canada; 5 Department of Pharmacology, Universitée de Sherbrooke, Sherbrooke, QC Canada; 6 Center for Research in Radiotherapy, Department of Nuclear Medicine and Radiobiology, Université de Sherbrooke, Sherbrooke, QC Canada; 7 Department of Pathology, Centre Hospitalier Universitaire de Sherbrooke, Sherbrooke, QC Canada

**Keywords:** Brain tumor, Carboplatin, Convection-enhanced delivery, Glioblastoma, Liposome

## Abstract

**Background:**

Effectiveness of chemotherapy for treating glioblastoma (GBM) brain tumors is hampered by the blood–brain barrier which limits the entry into the brain of most drugs from the blood. To bypass this barrier, convection-enhanced delivery (CED) was proposed to directly inject drugs in tumor. However, the benefit of CED may be hampered when drugs diffuse outside the tumor to then induce neurotoxicity. Encapsulation of drugs into liposome aims at increasing tumor cells specificity and reduces neurotoxicity. However, the most appropriate liposomal formulation to inject drugs into brain tumor by CED still remains to be determined. In this study, four liposomal carboplatin formulations were prepared and tested in vitro on F98 glioma cells and in Fischer rats carrying F98 tumor implanted in the brain. Impact of pegylation on liposomal surface and relevance of positive or negative charge were assessed.

**Results:**

The cationic non-pegylated (L1) and pegylated (L2) liposomes greatly improved the toxicity of carboplatin in vitro compared to free carboplatin, whereas only a modest improvement and even a reduction of efficiency were measured with the anionic non-pegylated (L3) and the pegylated (L4) liposomes. Conversely, only the L4 liposome significantly increased the median survival time of Fisher rats implanted with the F98 tumor, compared to free carboplatin. Neurotoxicity assays performed with the empty L4′ liposome showed that the lipid components of L4 were not toxic. These results suggest that the positive charge on liposomes L1 and L2, which is known to promote binding to cell membrane, facilitates carboplatin accumulation in cancer cells explaining their higher efficacy in vitro. Conversely, negatively charged and pegylated liposome (L4) seems to diffuse over a larger distance in the tumor, and consequently significantly increased the median survival time of the animals.

**Conclusions:**

Selection of the best liposomal formulation based on in vitro studies or animal model can result in contradictory conclusions. The negatively charged and pegylated liposome (L4) which was the less efficient formulation in vitro showed the best therapeutic effect in animal model of GBM. These results support that relevant animal model of GBM must be considered to determine the optimal physicochemical properties of liposomal formulations.

## Background

The efficacy of chemotherapeutic drugs to treat glioblastoma (GBM) is hampered by the blood–brain barrier (BBB), which limits drug accumulation in tumor cells [1]. Recently, chemotherapy drug delivery by convection-enhanced delivery (CED) [2] has attracted considerable attention. For example, CED of a liposomal formulation of irinotecan resulted in a better survival time than intravenous (i.v.) injection of the same drug in a GBM mouse model [3]. The efficiency of platinum compounds injected by CED was compared to different routes of delivery [i.v., intra-arterial (i.a.), or i.a. plus blood-brain barrier disruption] for their concomitant effect with radiotherapy in animal models of brain tumors. These results showed that CED of free platinum-based drugs increased the uptake of platinum in tumor and improved the median survival time of F98 glioma-bearing Fischer rats when combined with radiation [4–8]. Amongst the platinum compounds tested (carboplatin, cisplatin and oxaliplatin), carboplatin exhibited the lowest toxicity while providing the best survival benefits. In addition, induction of DNA damage by low energy secondary electrons produced by radiation, was greater when using carboplatin compared to cisplatin, suggesting that carboplatin is a better radiosensitizer [9]. These studies suggest that carboplatin is the best candidate among the approved platinum drugs for treatment of human brain tumors. In addition, a phase I clinical trial has shown that carboplatin delivered by CED is feasible and safe [10, 11].

Although CED increases the accumulation of carboplatin in tumor, its concentration decreases rapidly. For example, initially injected into the tumor at 600 µg/g of tissue, the concentration of carboplatin decreased to only 16 µg/g tissue in 24 h [4]. To improve its retention time in tumor and consequently increase the survival time of animals, liposomal formulations of carboplatin were suggested [12–14]. A negative or positive charge is needed on the liposomes to facilitate binding to charged cell membranes and to prevent clustering, which reduces their availability. However, the effects of liposome size, charge, and formulation on drug distribution volume after CED, still need to be optimized [15–20]. In our study, four different liposomal formulations of carboplatin were assessed: (1) cationic liposomes without pegylation (PEG) (L1); (2) cationic liposomes with pegylation (L2); (3) anionic liposomes without pegylation (L3); and (4) anionic liposomes with pegylation (L4). The addition of PEG to cationic liposomes should reduce their interaction with the negatively charged cell surface [21]. The distribution volume of pegylated liposomes is therefore expected to be increased, although the internalizing efficiency in F98 glioma cells may be reduced [18, 22, 23]. Since drug-free cationic liposomes could be neurotoxic, empty anionic liposomes with and without pegylation were also assessed [18, 24]. The in vitro toxicity of these liposomal carboplatin formulations was measured, as well as their maximum in vivo tolerated dose (MTD) and antitumor efficacy in a F98 glioma rat model.

## Results

### Characterization of liposomal carboplatin

**Fig. 1:**
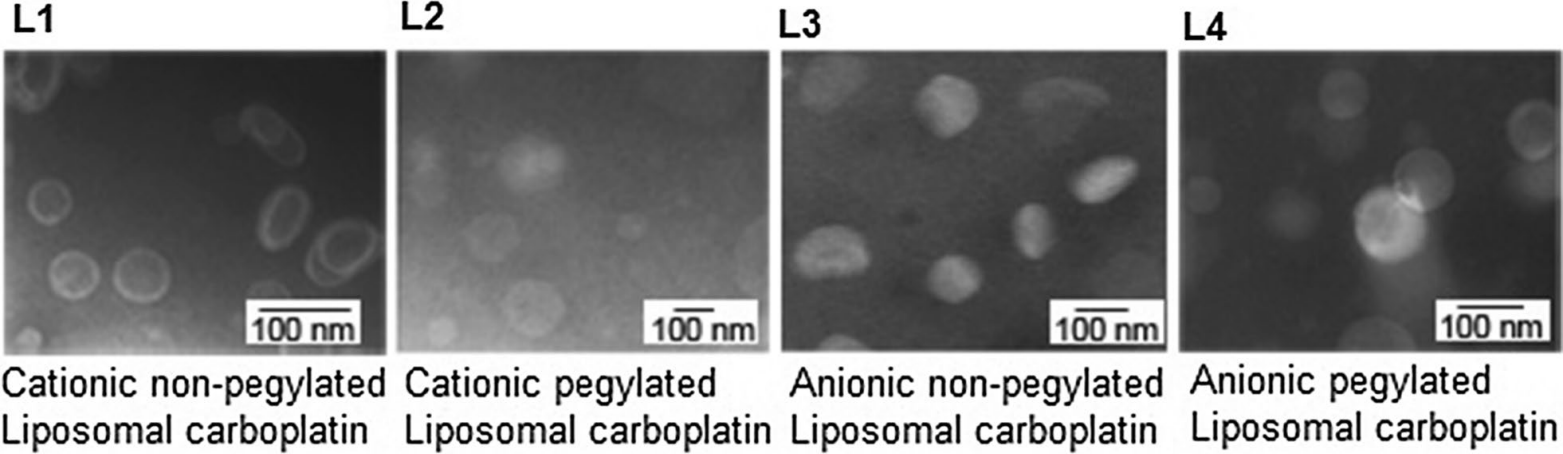
TEM images of negative stained unilaminar liposomes. Scale bar = 100 nm

The 4 types of liposomal carboplatin are detailed in Table 1: L1 = cationic non-pegylated liposomal carboplatin; L2 = cationic pegylated liposomal carboplatin; L3 = anionic non-pegylated liposomal carboplatin; and L4 = anionic pegylated liposomal carboplatin. The negative or positive charge was confirmed by measuring Zeta potentials and ranged between − 48.0 and 55.9 mV, consistant with values reported for similar liposomal formulations [25, 26]. Addition of 4.8% pegylation has only slightly reduced the Zeta potential values, as reported by Qin et al. [27]. Images of liposomes were captured under TEM with negative stain (Fig. 1). Unilaminar liposomes with sizes ranging from 57.4 to 85.6 nm were measured (Table 1).

**Table 1:** Properties of the liposomal formulation of carboplatin

ID	Chemical	Lipids molar ratio	Properties	Zeta potential (mV)	Method	Size (nm)	mg carboplatin mg lipids
L1	DPPC:DC-Chol	1:1	cationic	52.1 ± 8.84	REV	60.3 ± 21.1	0.050
L1′	DPPC:DC-Chol	1:1	cationic	55.9 ± 9.38	REV	57.4 ± 16.1	–
L1″	DPPC:DC-Chol	1:1	cationic	38.9 ± 12.9	Hydration	77.7 ± 21.0	–
L2	DPPC:DC-Chol: PEG2000 PE	10:11:1	cationic + PEG	45.1 ± 12.6	REV	71.3 ± 17.8	0.032
L3	DSPC:DSPG:Chol	7:2:1	anionic	− 48.0 ± 15.0	Hydration	72.7 ± 24.3	0.096
L3′	DSPC:DSPG:Chol	7:2:1	anionic	− 47.3 ± 14.6	Hydration	83.6 ± 17.2	–
L4	DPPC:Chol:PEG2000 PE	10:11:1	anionic + PEG	− 35.8 ± 5.43	REV	85.6 ± 19.9	0.32
L4′	DPPC:Chol:PEG2000 PE	10:11:1	anionic + PEG	− 33.0 ± 6.27	REV	77.4 ± 24.3	–

*PEG* pegylated liposomes, *REV* reverse-phase evaporation method, *Hydration* hydration method

For the cationic liposomal formulations L1 and L2, concentrations of 0.050 and 0.032 mg carboplatin per mg lipids were obtained. A greater accumulation of carboplatin was measured in the anionic formulation L3, which reached 0.096 mg per mg lipids. It is noteworthy that the liposomes L4 were by far the best at encapsulating carboplatin, reaching a concentration of 0.32 mg per mg lipids (Table 1).

### In vitro toxicity of drug-free liposomes and liposomal carboplatin

**Fig. 2:**
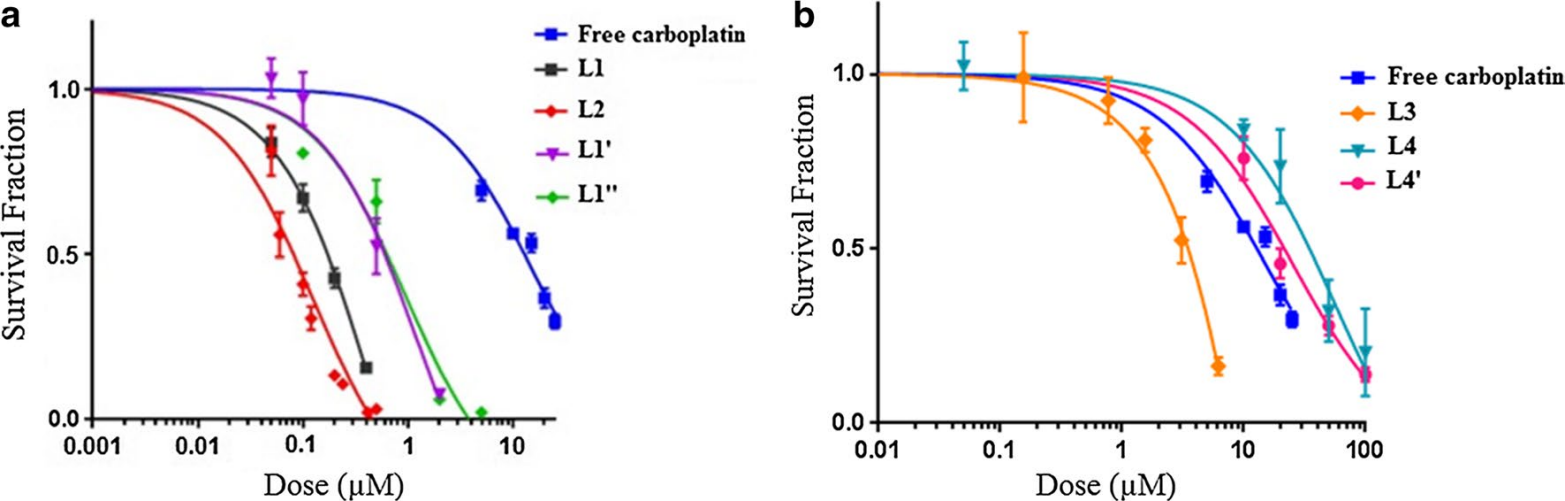
Survival of F98 cells after 24 h incubation with free carboplatin, liposomal carboplatin or drug-free liposomes. **a** LD50: free carboplatin = 13.6 µM; L1 = 0.169 µM; L2 = 0.088 µM; L1′ = 6.07 µM*; L1″ = 8.08 µM*. **b** LD50: L3 = 3.33 µM; L4 = 35.0 µM; L4′ = 32.7 µM*. *The LD50 for the empty liposomes L1′, L1″ and L4′ are reported as lipid concentration

Toxicity associated with the different liposomal formulations on the F98 cells were determined (Fig. 2, Table 2). The 2 cationic liposomal carboplatin showed higher toxicity than the anionic formulations. A LD50 of 0.169 µM carboplatin was measured with the non-pegylated liposomal carboplatin L1. Encapsulation of carboplatin in this cationic liposomal formulation decreased its efficiency, leading to a LD50 80 times higher than that of free carboplatin (LD50 = 13.6 µM). The drug-free cationic non-pegylated liposome L1′ showed some toxicity for the F98 cells, with an LD50 of 6.07 µM in lipid equivalent, which corresponds to about 3 times more lipids then used for the carboplatin formulation L1 (Fig. 2a, Table 2). To determine whether this toxicity was caused by the preparation method, the same liposomal formulation was prepared by the hydration method (L1″). The toxicity of liposomes L1″ (LD50 of 8.08 µM total lipids) on the F98 cells, was similar to that measured with L1′. At the LD50 of liposomal carboplatin L1, the total lipids concentration was 1.98 µM, which corresponded to about 3.6 times less lipids than at the LD50 concentrations of formulations L1′ and L1″. Therefore, while it cannot be excluded that a part of the toxicity measured with the carboplatin formulation L1 is due its lipid components, the toxic effect on the F98 cells was mainly caused by carboplatin.

**Table 2:** LD50 and cellular uptake of free carboplatin and liposomal formulations of carboplatin

ID	Drugs	LD50		Cellular uptake^a^ (ng Pt/10^6^ cells)	Cellular uptake normalized for LD50 (ng Pt/10^6^ cells/µM LD50)
		Carboplatin (µM)	Lipids (µM)		
	Dextrose	–	–	–	–
	Carboplatin	13.6	–	12.8 ± 1.2	0.94
L1	Liposomal carboplatin	0.169	1.98	36.8 ± 8.0	218.75
L1′	Hollow liposomes	–	6.07	–	–
L1″	Hollow liposomes	–	8.08	–	–
L2	Liposomal carboplatin	0.088	1.03	1.9 ± 0.1	21.64
L3	Liposomal carboplatin	3.33	17.0	8.7 ± 2.1	2.61
L3′	Hollow liposomes	–	> 509	–	–
L4	Liposomal carboplatin	35.0	30.8	26.5 ± 1.9	0.76
L4′	Hollow liposomes	–	32.7	–	–

^a^Cell uptake of carboplatin (Pt) measured after 24 h incubation at the concentration corresponding to the LD50 of each drug

The second cationic formulation L2 was prepared by adding ~ 5 mol% PEG2000 PE (Table 1). This cationic pegylated liposome exhibited a slightly, but not significantly, lower positive charge, which was associated with the use of the anionic PEG2000 PE. It is noteworthy that the addition of PEG-modified lipids improved the anti-cancer potential of carboplatin encapsulated in this liposomal formulation, as shown by its lower LD50 (0.088 µM) compared to 0.169 µM for the L1 liposome.

The anionic liposome L3 was made with 20 mol% DSPG, an anionic lipid that is known to be a suitable replacement for PEG-modified lipids in terms of reducing surface–surface association, which can lead to liposome aggregation and/or liposome-cell interactions [27, 28]. The LD50 of this formulation prepared without carboplatin (L3′) was more than 500 µM total lipids, making it by far the least toxic of the cationic formulations tested. When carboplatin was encapsulated in this anionic formulation L3, a large improvement in LD50 was measured, reaching 3.33 µM carboplatin, which corresponded to 17.0 µM total lipids. These results also indicate that the toxicity of L3 on F98 cells was due solely to the carboplatin. Furthermore, the L3 formulation was about 4-times more efficient than free carboplatin (LD50 = 13.6 µM).

For the last formulation L4, DC-Chol was replaced by cholesterol to remove the positive charge of the liposomes, while PEG2000 PE was included to obtain a negative Zeta potential (Table 1). These L4′ anionic liposomes without carboplatin shown a LD50 of 32.7 µM which was more than five times less toxic than the cationic liposomes without carboplatin L1′ and L1″. However, encapsulation of carboplatin in the anionic pegylated formulation L4 was not effective for treating the F98 cells since its LD50 was 35.0 µM, which corresponded to 30.8 µM of lipids used to make the liposomes. These results suggested that the toxicity of the L4 may be caused by the lipids used to make them. Moreover, the efficiency of the L4 liposome was 10.5 to 400 times lower than the other liposomal formulations of carboplatin (Table 2).

### Cellular accumulation of liposomal formulations carboplatin

Drug delivery to cancer cells can be affected by the presence of negative or positive charge and also of PEG on the liposome surface [18]. The ability of our liposomal formulations to deliver carboplatin to the F98 cells was therefore determined experimentally. Cellular carboplatin concentration was normalized to take account of the LD50 of each liposomal formulation. The cationic non-pegylated L1 increased by more than 230 times the carboplatin concentration in the F98 cells relative to free carboplatin. The addition of PEG on the cationic liposome (L2) decreased by 10 times the concentration of carboplatin in the F98 cells relative to the non-pegylated L1, but it still resulted in a 23 times higher accumulation than free carboplatin. On the other hand, only a marginal improvement of carboplatin uptake was measured with the anionic liposome L3, while the pegylated anionic liposome L4 didn’t lead to any improvement relative to free carboplatin.

### Median survival time (MeST) of F98 glioma bearing rats treated with liposomal carboplatin

The MTD of cationic non-pegylated liposomal carboplatin (L1, 10 µg) and the pegylated one (L2, ≥ 18 µg, maximum injectable dose) were lower than achieved with free carboplatin (25 µg), which showed that these cationic formulations couldn’t reduce the toxicity of carboplatin on Fisher rats (Table 3). Conversely, the anionic liposomes successfully reduced the toxicity of carboplatin. The liposomal carboplatin L3 and the pegylated form L4 exhibited a MTD of > 38.7 µg and 50 µg, respectively.

**Table 3:** Median survival time of F98 glioma bearing Fischer rats

ID	Drugs	MTD (µg)	MeST (days)	Range
	Dextrose	–	23.5	20–25
	Carboplatin	25	38.5	31–47
L1	Liposomal carboplatin	10	35.0	31–42
L1′	Hollow liposomes	–	22.5	22–23
L1″	Hollow liposomes	–	–	
L2	Liposomal carboplatin	18^a^	29.0	25–32
L3	Liposomal carboplatin	38.7^a^	31.0	28–34
L3′	Hollow liposomes	–	–	
L4	Liposomal carboplatin	50	49.5	31–71
L4′	Hollow liposomes	–	25	22–26

*LD50* lethal dose for 50% of the cells, *MeST* Median survival time, *MTD* maximum tolerated dose

^a^Maximal concentration that can be injected

**Fig. 3:**
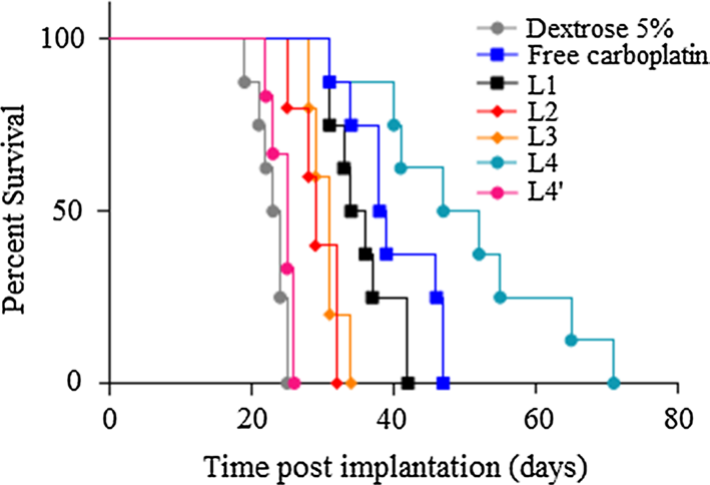
Kaplan–Meier survival curve of F98 glioma bearing rats treated with CED of different carboplatin formulations. Cationic liposomes L1 and L2 improved median survival times (35 days for non-pegylated L1, 29 days for pegylated L2) when compared to control (dextrose 5%) (23.5 days), but shorter or equivalent survival time when compared to free carboplatin (38.5 days). Anionic pegylated liposomal carboplatin L4 offered the best median survival time (49.5 days) and showed a better median survival time than free carboplatin. Injection with the empty anionic pegylated liposome L4′ didn’t improved the median survival time (25 days) which was similar to animals injected with dextrose 5%. Log Rank test *p* value compared to 5% dextrose: L1, *p* < 0.0001; L2, *p *= 0.0018; L3, *p *= 0.0003; L4, *p* < 0.0001; L4′, *p* = 0.447 (*p* = 0.0001, when compared to L4), free carboplatin, *p* < 0.0001

Their anti-tumor efficiency were then assessed at their respective MTD, or at the maximum injectable dose for L2 and L3, in Fischer rat bearing the F98 GBM tumor (Fig. 3, Table 3) to comply with clinical practice. Free carboplatin increased animal survival time by 15 days with respect to the untreated group (carboplatin = 38.5 days; untreated group = 23.5 days, *p* < 0.0001). Encapsulation of carboplatin in the liposomes L1 didn’t significantly increase the MeST, which reached 35 days (*p* value, free carboplatin vs L1 = 0.08). The addition of PEG was no benefit since a reduction in anti-tumor activity relative to free carboplatin was measured for the cationic pegylated liposomal L2, as shown by a shorter MeST of 29 days (*p* value, free carboplatin vs L2 = 0.0015). The anionic non-pegylated liposome L3 was no better with a MeST of 31 days (*p* value, L2 vs L3 = 0.6313). It is noteworthy that L2 and L3 were not assessed at their MTD but instead at their maximum injectable dose.

The highest anti-tumor efficiency was observed with the anionic pegylated liposomal carboplatin L4, with a MeST of 49.5 days, which is significantly better than that achieved with free carboplatin (*p* = 0.0335), and the cationic liposome L1 (*p* = 0.0008). The empty liposomes L4′ (carboplatin free) showed a similar MeST to that of the untreated group, which suggests that the lipid formulation of L4 was not itself toxic to tumor cells.

### Accumulation of carboplatin in brain tumor

**Fig. 4:**
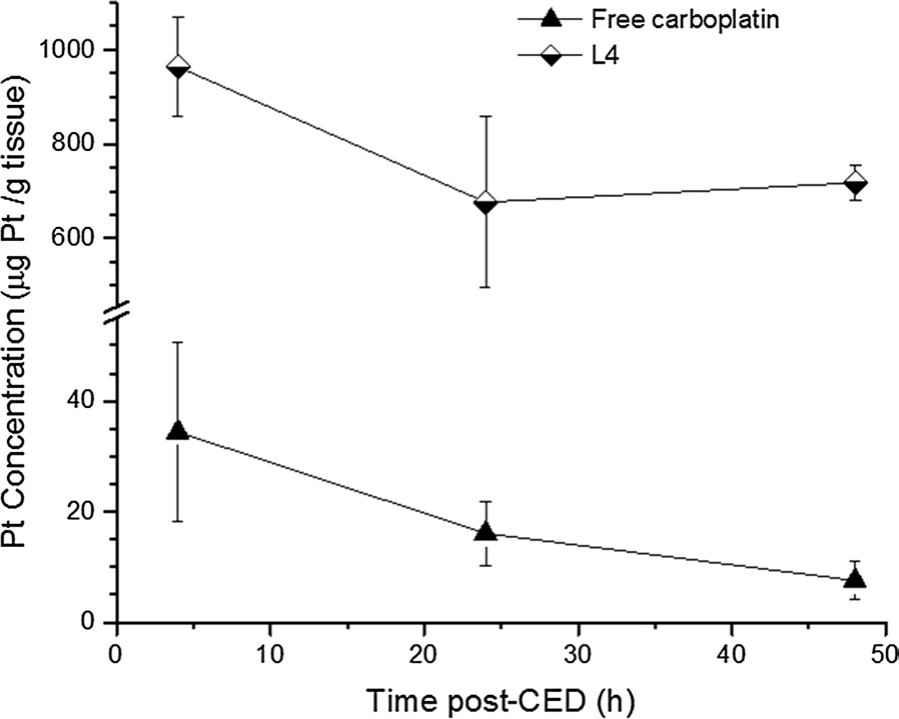
Tumor retention of anionic pegylated liposomal carboplatin (L4) and free carboplatin over a period of 48 h

The ability of L4 liposomes, which achieved the best therapeutic outcomes in the F98 model, to deliver carboplatin in brain tumor by CED was determined. The amount of carboplatin in tumor was quantified at 4, 24 and 48 h after CED by ICP-MS. Free-carboplatin injected at its MTD was rapidly eliminated (Fig. 4). Four hours after its injection, the carboplatin concentration in tumor reached only 34.4 µg/g tissue. This rapid elimination of carboplatin was largely prevented by its encapsulation into the liposome L4 which allowed a concentration of 963.7 µg/g tissue to be maintained at 4 h after CED (Fig. 4). Furthermore, the concentration of carboplatin in tumor has decreased by only 1.3-fold at 48 h after CED, reaching 719.1 µg/g tumor. These results indicate that the anionic liposomal formulation L4 favors the accumulation and retention of carboplatin in the brain tumor F98.

### Toxicity of empty liposome L4′

**Fig. 5:**
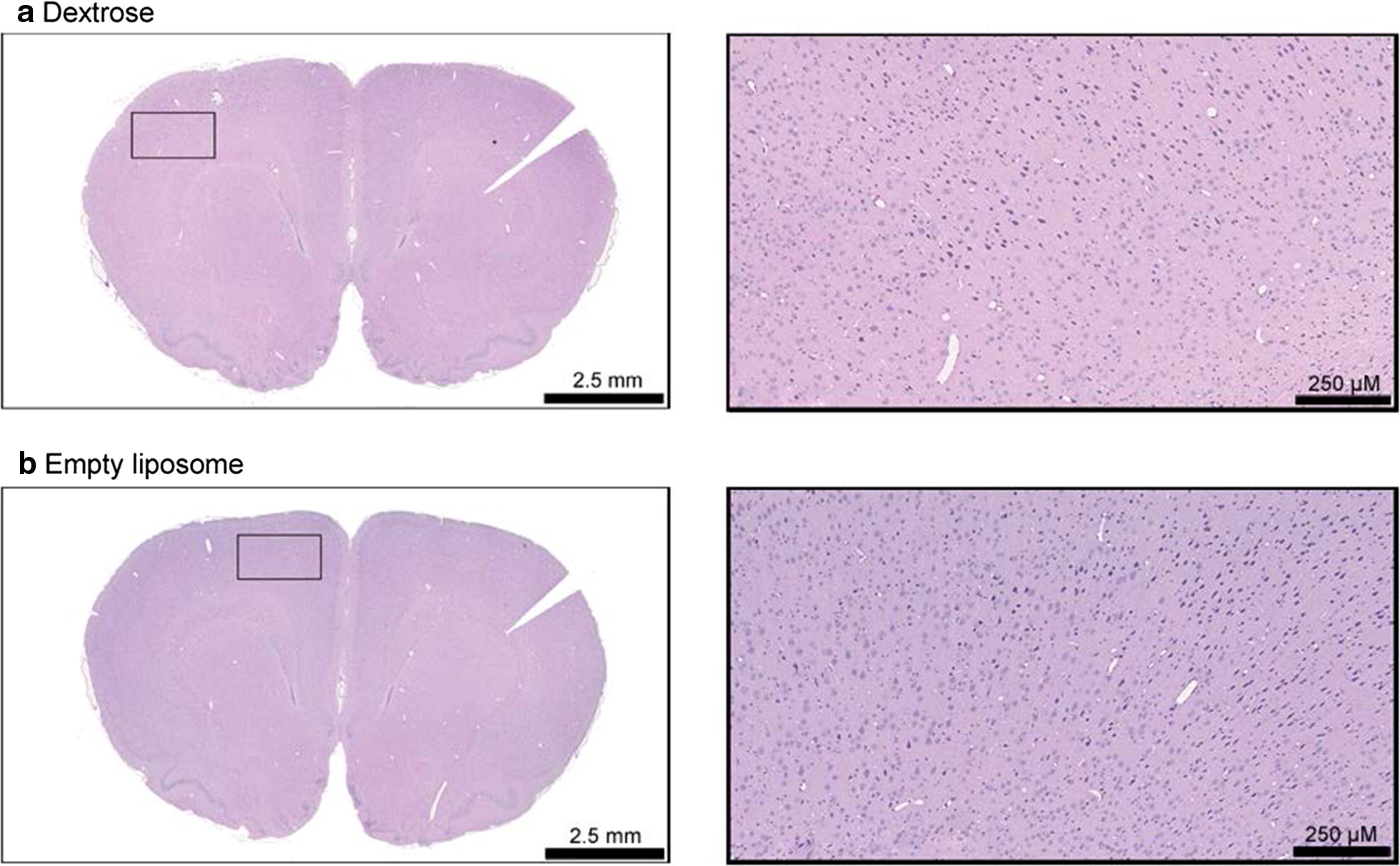
Neurotoxcity assessment of L4′ liposome. Dextrose (**a**) or liposome L4′ (**b**) was injected in tumor-free animals. The liposome L4′ was administered at a lipid concentration equivalent to that of the MTD of the liposome L4. Fifty days post-CED, the histopathological analyses showed that the liposome L4′ didn’t affect brain structures, didn’t activate microglia, and didn’t induce necrosis

The empty liposome L4 (named L4′) was administrated by CED to assess the contribution of its lipid components in the improvement of MeST in rat implanted with the F98 tumor, and also in tumor-free animals to assess its neurotoxicity. The liposome L4′ was injected at a lipid concentration equivalent to that of the MTD of the liposome L4. In animal implanted with the F98 tumor, the liposome L4′ didn’t modify the MeST which shows that this empty liposome had no therapeutic effect on the tumor cells (Table 3, Fig. 3). Fifty days post-CED in tumor-free animals, no signs of neurotoxicity were observed. Histopathological analyses showed that the empty liposome L4′ didn’t affect brain structures (Fig. 5). These neurotoxic analyzes were not performed with the other liposomal formulations since they did not improve the MeST compared to that of free carboplatin.

## Discussion

To overcome this obstacle of BBB, platinum-based drugs have been injected directly into the tumor by CED in animals bearing a glioblastoma [4, 6–8, 29]. The pressure gradient created by CED should significantly increase the drug distribution volume in the brain allowing higher concentrations to be attained over longer distances [28].

Carboplatin was chosen for our study, because it is better tolerated than cisplatin and oxaliplatin, and produces the highest therapeutic benefit in GBM animal models [4–8]. However, one shortcoming of carboplatin delivery via CED is its rapid clearance from the extracellular compartment. To overcome this limitation, liposomal formulations were developped to enhance tumor retention and reduce neurotoxicity. Physicochemical properties of liposomes can influence their ability to retain a drug, bind to target cells, and spread through a tumor [28, 30, 31]. In our study, the effects of charge and PEG on four liposomal formulations of carboplatin were assessed from: (i) a cationic non-pegylated formulation (L1), (ii) a cationic pegylated formulation (L2), (iii) a anionic non-pegylated formulation (L3), and (iv) a anionic pegylated formulation (L4).

Bypassing the BBB with a direct injection into the tumor enables the concentration of carboplatin to exceed that obtainable by i.v. injection. Using the same animal model of GBM, systemic injection of liposomal formulations of cisplatin or oxalyplatin led to only 0.32 µg/g tissue and 0.14 µg/g tissue of these platinum derivatives in tumor [5], which corresponds to 3000 to 7000 times lower concentrations than obtained after CED injection with our liposome L4. These results also suggest that liposome L4 administrated by CED may circumvent carboplatin resistance in cancer cells.

After distribution of the liposomes through the tumor by CED, the restricted extracellular space may improve their retention. In our study, encapsulation of carboplatin in pegylated liposome L4 has increased by 28 times the concentration of this platinum derivative in F98 tumor, relative to free carboplatin. The clerance rate between the period of 4 h to 48 h post-CED was also significantly reduced with the liposome L4. The concentration of free carboplatin decreased by 4.5 times, while those of liposome L4 went down by only a factor 1.3. Using the same animal model, a similarly rapid elimination of free carboplatin after its injection directly in the tumor was reported, as only 10.4 µg/g tissue was measured immediately after CED [8].

When a drug is injected directly into the brain, minimizing its neurotoxicity becomes a major issue. Drug encapsulation into liposomes was thus initially proposed to reduce the neurotoxicity [18, 26, 32]. However, some liposomal formulation can be very neurotoxic. The negatively charged Lipoplatin™ and its empty version without cisplatin were highly neurotoxic when given by CED in rat brain resulting in death immediately following or within a few days after administration [26]. Intracranial injection of liposomes without drug, composed of lecithin–cholesterol–dicetyl phosphate (net negative charge) or lecithin–cholesterol–stearylamine (net positive charge) induced epileptic seizures and some deaths due to respiratory failure immediately after injection, and a subsequent widespread tissue necrosis [33]. Assessed in tumor free animals, our cationic liposomal formulation L1 also failed to reduce the neurotoxicity as its MTD was even lower than free carboplatin. The MTD of the pegylated version L2, couldn’t be assessed as the intracranial injection was limited by maximal injectable volume. The 2 negatively charged liposomes L3 and the pegylated L4 were better tolerated as their MTD was about 2-times higher than that of free carboplatin. The neurotoxicity could be caused either by the lipid composition of the liposome, or be associated to the release of carboplatin from the liposomes into the brain. Consequently, the potential neurotoxicity of the empty liposomes L4 (named L4′) was assessed at a lipid concentration equivalent to that of the MTD of the liposomes L4. Fifty days post-CED, no signs of neurotoxicity were observed. Histopathological analyses showed that the empty liposomes L4′ didn’t affect brain structures. In animal implanted with the F98 tumor, the liposome L4′ didn’t modify the MeST of the animals, which shows that this empty liposomal formulation had no therapeutic effect on the tumor cells nor significant neurotoxicity.

Another benefit of adding PEG is to increase the stability of drug encapsulation in liposomes, which can potentially reduce its toxicity to non-target cells [34–36]. Although a significant improvement is already measured with only 4% PEG, a much higher stability can be achieved with 8% PEG and more [35]. Since our liposomes contained 4.8% PEG, this addition may have contributed to reduce carboplatin toxicity for cerebral parenchyma in our rat model.

Lipid composition, charge, pegylation, and diameter of liposomes and also the chemical characteristics of a drug affect its loading efficiency into liposomes [37]. With our liposomal formulations, the highest encapsulation of carboplatin was obtained with the cationic pegylated formulation L4, which reached a concentration similar to that previously reported by Zhang et al. [38]. Our results also support the hypothesis that higher drug loading can be obtained with positive liposome compared to those with a negative charge [39, 40], which can be further increased by adding PEG [40].

Carboplatin and its analogues cisplatin and oxaliplatin use the copper transporter Ctr1 to cross the cell membrane. Resistance to cisplatin has been associated with a reduction in the efficacy of this receptor [41]. Liposomes are taken up by the tumor cells either via phagocytosis or by direct fusion with the cell membrane. These two mechanisms enable a 10- to 400-fold higher intracellular uptake of platinum drugs in tumor cells compared to normal cells, by bypassing the carboplatin resistance of tumor cells [42]. It was also shown that the uptake of cationic liposomes by cancer cells was about 20-fold higher than for anionic liposomes [43]. We have measured a similar enhancement of carboplatin accumulation in vitro in the F98 cells. The highest accumulations of carboplatin were obtained with the cationic liposomes L1 and L2, while a modest increase was measured with the anionic liposomes L3. Pegylation of the anionic liposome reduced the net negative charge of the liposome L4, and this modification seems to have reduced the interaction between the liposome and the cancer cells, since no improvement of carboplatin accumulation in the F98 cells occured. It was previously suggested that PEG can reduce the uptake of liposome by cells, because the binding of these polymers to cell membranes decreases the cell surface hydrophobicity, which reduces the fluid phase of endocytic process [44], and consequently compromises the internalization of liposome [18, 31].

The relative effectiveness of our liposomes to increase carboplatin accumulation corresponds exactly to their ability to eliminate the F98 cells in vitro, the best one being the best the L1 formulation. Conversely, the liposome L4 that couldn’t improved the LD50 of carboplatin in vitro, was surprisingly the only liposome formulation to increase significantly the MeST of rats implanted with the F98 tumor.

We hypothesise that the higher therapeutic efficiency of our pegylated anionic liposome L4 compared to the cationic liposomes L1 and L2 was caused by a larger distribution volume in the tumor. Indeed, Mackay et al. have reported that their anionic 80 nm liposomes travelled for longer distances as they obtained a penetration radius of about 0.8 mm from the CED injection site, compared to less than 0.2 mm for their cationic liposomes [18]. Similar observations were made by Nie et al. who reported that their cationic liposomes were more readily taken up by cells, both in vitro and in vivo, because of the interaction with the negatively-charged cell membrane [45], which can thus contribute to reduce the distribution volume of cationic liposomes.

When delivered by CED, pegylated liposomes could travel for longer distances than non-pegylated liposomes. Presumably, steric shielding caused by PEG reduces the rate of liposome binding to cells in the brain during CED [18]. However, MacKay et al. have reported that a significant quantity of their pegylated and non-pegylated cationic liposomes were located near the infusion cannula used for CED [18]. The tumor cells near the injection site were thus exposed to a higher concentration of carboplatin than those located farther within the tumor. This non-uniform distribution of carboplatin could explain why our cationic liposomes did not significantly improve MeST of the animals.

## Conclusions

The in vitro and in vivo behaviors of our cationic and anionic liposomes and their therapeutic efficiency varied drastically. Cationic liposomes are known to bind more efficiently to the cell membrane, facilitating carboplatin accumulation in tumor cells and increasing their therapeutic efficacy in vitro. In the animal model of GBM, this property of cationic liposomes didn’t significantly improve the MeST. Conversely, the anionic and pegylated liposomes can diffuse over a larger distance in the tumor, probably resulting in a more uniform distribution of carboplatin. Supporting this hypothesis, our anionic pegylated liposome L4 was the best to improve the MeST, and the encapsulation of carboplatin in this liposome had also the advantage of increasing its concentration in the tumor, reducing its clearance rate and reducing neurotoxicity relative to free carboplatin. With these properties and an anti-tumor efficiency significantly better than that achieved with free carboplatin, intratumor injection of liposomal carboplatin L4 should be considered as a promising replacement for pure carboplatin in the chemotherapeutic treatment of GBM tumors.

## Methods

### Chemicals

Carboplatin was purchased from Hospira (Montreal, Canada) and Hande Tech Development Co (Houston, USA). 1,2-dipalmitoyl-sn-glycero-3-phosphocholine (DPPC), cholesterol (Chol), 3ß-[*N*-(*N*′,*N*′-dimethylaminoethane)-carbamoyl]cholesterol hydrochloride (DC-Chol), 1,2-dipalmitoyl-sn-glycero-3-phosphoethanolamine-*N*-[methoxy(polyethylene glycol)-2000] (PEG2000 PE), 1,2-distearoyl-sn-glycero-3-phosphocholine (DSPC), and 1,2-distearoyl-sn-glycero-3-[phospho-rac-(1-glycerol)] (sodium salt) (DSPG) were obtained from Avanti Polar Lipids Inc (Alabaster, USA).

### Preparation of liposomal carboplatin

The liposomal formulations of carboplatin identified as cationic non-pegylated (L1), cationic pegylated (L2), and anionic pegylated (L4), and also the carboplatin-free liposomes cationic non-pegylated (L1′), and anionic pegylated (L4′) were prepared by the reverse phase evaporation method [46]. Briefly, DPPC:DC-Chol (1:1 mol) (for cationic non-pegylated liposomes) or DPPC:DC-Chol/Chol:PEG2000 PE (10:11:1 mol) (for cationic/anionic pegylated liposomes) were co-dissolved in chloroform:isopropyl ether (1:1 vol) solvent. Then, 2 mL of 27 mM carboplatin or 5% dextrose (for empty liposomes) was added and sonicated for 5 min. The organic solvent was removed under reduced pressure for 15 min by a rotary evaporator. Rotary evaporation was then continued for 1 h. Then, unencapsulated carboplatin was removed by PD-10 desalting column containing Sephadex™ G-25 Medium (GE Healthcare Bio-Sciences Corp, Piscataway, USA).

Anionic non-pegylated liposomal carboplatin (L3) and carboplatin-free anionic non-pegylated liposomes (L3′) were prepared by the hydration method through dissolving DSPC:DSPG:Chol (7:2:1 mol) in chloroform:methanol:water (50:10:1 vol). Lipids were then dried under a stream of nitrogen gas to form a thin film. Subsequently, the lipid film was subjected to low vacuum to remove any residual solvent. Lipid films were then hydrated by vortexing in the presence of hot (70 °C) SH buffer (300 mM sucrose, 20 mM HEPES, pH 7.5) with or without carboplatin (100 mM). The resulting lipid-buffer suspension was then extruded 10 times through stacked 100 nm and 80 nm polycarbonate filters at 70 °C (Northern Lipids Inc., BC, Canada). The external buffer was then exchanged with SH buffer at room temperature by a G-50 Sepadex™ column. The concentration of carboplatin was determined by inductively coupled plasma mass spectrometry (ICP-MS) (Thermo Scientific XSERIES 2, Thermo Fisher Scientific Inc.) [32].

As an additional control, empty cationic non-pegylated liposomes (L1″) were also prepared according to the hydration method by dissolving DPPC:DC-Chol (1:1 mol) in chloroform:isopropyl ether (1:1 vol) solvent. Lipids were then dried under reduced pressure by a rotary evaporator to form a thin film. Lipid film was then hydrated by vortexing in the presence of 2 mL 5% dextrose. The resulting lipid-buffer suspension was frozen and thawed for five times, and then extruded 10 times through stacked 100 nm and 80 nm polycarbonate filters at 50 °C (Northern Lipids Inc., BC, Canada).

### Characterization of liposomal carboplatin

The size and appearance of liposomes containing carboplatin were characterized with a Hitachi H-7500 transmission electron microscopy (TEM) operating at 80 kV. Samples were negative stained by mixing liposomal carboplatin with ammonium heptamolybdate and 2% of uranyl acetate. Charge on the liposomes was confirmed by measuring the Zeta-potential with a zetasizer nano ZS (Malvern Instruments Ltd., Worcestershire, UK). Phospholipids were quantified by choline oxidase-DAOS method following the protocol of Wako Phospholipids C (Wako Diagnostics Wako Chemicals USA, Inc., Richmond, USA).

### Cell lines and animal model

F98 rat glioblastoma cells were purchased from American Type Culture Collection. Male Fischer rats of 210–225 g were purchased from Charles River Laboratories (Saint-Constant, Canada). The experimental animal protocol was approved by the institutional ethical committee and complied with the regulations of the Canadian Council on Animal Care (protocol # 329-13B).

### Cytotoxicity study of carboplatin and its liposomal formulations in F98 cells

Cytotoxicity was assessed using a clonogenic assay as previously described [47]. Briefly, 400 cells were plated in 6-well plate, and 24 h later carboplatin or its liposomal formulations in FBS-free DMEM medium were added. After an additional 24 h, the drug-containing medium was removed, the cells were washed, and new DMEM medium enriched with 10% FBS, was added. Seven days later the colonies were stained with 0.1% crystal violet, and counted. The median lethal dose (LD50) for each liposomal formulation of carboplatin was then determined.

### Cellular uptake of carboplatin and its liposomal formulations in F98 cells

F98 cells were incubated with carboplatin or one of the liposomal formulations at their respective LD50 for 24 h in FBS-free DMEM medium, and then washed three times with PBS, harvested, and counted. Cells were digested in 70% HNO_3_ and 30% H_2_O_2_, followed by quantification for platinum with Inductively Coupled Plasma Mass Spectrometry (ICP-MS) (ELAN DRC-II, PerkinElmer) [32]. Data were expressed as ng of platinum per 10^6^ cells.

### F98 cells implantation in Fischer rat brain

As described previously [48], the rat’s head was mounted on a stereotactic frame, an incision was made in the middle of the head and the bregma exposed. A burr hole was made with a dental drill, 3 mm to the right, 1 mm anterior of the bregma. Five microliter of FBS-free DMEM containing 10,000 cells was injected 6 mm deep into the burr hole over 5 min at a constant flow. Then, the needle was slowly withdrawn over 1 min and the burr was sealed by bone wax. Finally, the incision was sutured and smeared with antibiotic cream.

### CED procedure

A solution of free carboplatin or liposomal formulations (10 µL in 5% dextrose) was injected by CED 10 days after tumor cell implantation with a microinfusion pump (World Precision Instruments) a rate of 0.5 µL/min for 20 min using a Hamilton syringe connected to a 33 Ga needle [4].

### Determination of MTD for the liposomal carboplatin

The MTD of liposomal carboplatin was determined in Fisher rats by the traditional 3 + 3 design, which consists of dose escalation and de-escalation based on the number of dose limiting toxicities observed in a cohort of 3 animals [49]. Rats were followed during 10 days after CED. Those that were unable to feed or groom, or lethargic were considered to be moribund and were euthanized.

### Assessment of median survival time

To comply with clinical practice, anti-tumor efficiencies were determined by animal survival assays performed at MTD. Ten days after F98 tumor implantation, each liposomal formulation and free carboplatin were injected by CED at their respective MTD. Animals were daily monitored which included weight measurement, mobility, coordination, loss of self-grooming (periocular secretion accumulation) and landing ability. They were euthanized when one of the monitored indexes reached a score of 1/10 or when they lost > 30% of their initial weight.

### Tumor uptake of free carboplatin and the anionic pegylated liposomal formulation L4

Free carboplatin or anionic pegylated liposomal carboplatin (L4) was delivered at their respective MTD by CED. Animals (n = 3) were euthanized at 4, 24 or 48 h after CED. Blood was evacuated by intra-cardiac infusion of 4% paraformaldehyde, and then the brain was extracted. Using a brain matrix, a slice of the brain was obtained from a millimeter before the tumor cell implantation point to a millimeter afterwards. Tumor and normal tissue around it were isolated, weighed, and digested in 70% HNO_3_ and 30% H_2_O_2_. The concentrations of platinum were analyzed by ICP-MS [32].

### Histopathology

Euthanasia was carried out by exsanguination and intra-cardiac perfusion of 30 ml formaldehyde 4% for histological analysis. Brain specimens were removed and kept in formaldehyde for 48 h prior to be cut in the coronal plane using a brain matrix (taking the implantation needle mark on the cortex as a reference point for the slicing), and finally embedded into paraffin. The blocks were cut into 5 μm thick slides and stained with haematoxylin and eosin.

### Statistical analysis

Results were analyzed by a Student’s *t* test to compare two treatments together and by ANOVA for more than two groups. The median survival times were determined by the Quartile method before doing Kaplan–Meier survival curves which were analyzed by Log-Rank test with GraphPad Prism software. A *p* value < 0.05 was considered as statistically significant.

## Abbreviations

BBB: blood–brain barrier
CED: convection-enhanced delivery
Chol: cholesterol
DC-Chol: 3ß-[*N*-(*N*’,*N*’-dimethylaminoethane)-carbamoyl]cholesterol hydrochloride
DPPC: 1,2-dipalmitoyl-sn-glycero-3-phosphocholine
DSPC: 1,2-distearoyl-sn-glycero-3-phosphocholine
DSPG: 1,2-distearoyl-sn-glycero-3-[phospho-rac-(1-glycerol)] (sodium salt)
GBM: glioblastoma
i.a.: intra-arterial
ICP-MS: inductively coupled plasma mass spectrometry
i.v.: intravenous
L1: cationic liposomes without pegylation
L2: cationic liposomes with pegylation
L3: anionic liposomes without pegylation
L4: anionic liposomes with pegylation
LD50: median lethal dose
MeST: median survival time
MTD: maximum in vivo tolerated dose
PEG: pegylation
PEG2000 PE: 1,2-dipalmitoyl-sn-glycero-3-phosphoethanolamine-*N*-[methoxy(polyethylene glycol)-2000]
TEM: transmission electron microscopy
